# Clinicians’ perspectives on inertial measurement units in clinical practice

**DOI:** 10.1371/journal.pone.0241922

**Published:** 2020-11-13

**Authors:** François Routhier, Noémie C. Duclos, Émilie Lacroix, Josiane Lettre, Elizabeth Turcotte, Nathalie Hamel, François Michaud, Cyril Duclos, Philippe S. Archambault, Laurent J. Bouyer

**Affiliations:** 1 Department of Rehabilitation, Université Laval, Quebec City, QC, Canada; 2 Center for Interdisciplinary Research in Rehabilitation and Social Integration, Centre Intégré Universitaire de Santé et de Services Sociaux de la Capitale-Nationale, Quebec City, QC, Canada; 3 Handicap Activity Cognition Health Team-U1219 BPH, University of Bordeaux, Bordeaux, France; 4 Institut Universitaire des Sciences de la Réadaptation, University of Bordeaux, Bordeaux, France; 5 IntRoLab–Laboratoire de Robotique Intelligente/Interactive/Intégrée/Interdisciplinaire, Institut Interdisciplinaire d’Innovation Technologique, Université de Sherbrooke, Sherbrooke, QC, Canada; 6 Department of Electrical Engineering and Computer Engineering, Université de Sherbrooke, Sherbrooke, QC, Canada; 7 School of Rehabilitation, Université de Montréal, Montreal, QC, Canada; 8 Centre for Interdisciplinary Research in Rehabilitation of Greater Montreal, Institut Universitaire sur la Réadaptation en Déficience Physique de Montréal, Centre Intégré Universitaire de Santé et de Services Sociaux du Centre-Sud-de-l'Île-de-Montréal, Montreal, QC, Canada; 9 School of Physical and Occupational Therapy, McGill University, Montreal, QC, Canada; 10 Centre for Interdisciplinary Research in Rehabilitation of Greater Montreal, Jewish Rehabilitation Hospital, Centre Intégré de Santé et de Services Sociaux de Laval, Laval, QC, Canada; Fondazione Ugo Bordoni, ITALY

## Abstract

Inertial measurement units (IMUs) have been increasingly popular in rehabilitation research. However, despite their accessibility and potential advantages, their uptake and acceptance by health professionals remain a big challenge. The development of an IMU-based clinical tool must bring together engineers, researchers and clinicians. This study is part of a developmental process with the investigation of clinicians’ perspectives about IMUs. Clinicians from four rehabilitation centers were invited to a 30-minute presentation on IMUs. Then, two one-hour focus groups were conducted with volunteer clinicians in each rehabilitation center on: 1) IMUs and their clinical usefulness, and 2) IMUs data analysis and visualization interface. Fifteen clinicians took part in the first focus groups. They expressed their thoughts on: 1) categories of variables that would be useful to measure with IMUs in clinical practice, and 2) desired characteristics of the IMUs. Twenty-three clinicians participated to the second focus groups, discussing: 1) functionalities, 2) display options, 3) clinical data reported and associated information, and 4) data collection duration. Potential influence of IMUs on clinical practice and added value were discussed in both focus groups. Clinicians expressed positive opinions about the use of IMUs, but their expectations were high before considering using IMUs in their practice.

## Introduction

The use of technology is constantly increasing in our daily lives and also in clinical settings, making patients’ assessment, therapy and follow-ups potentially easier and faster for healthcare professionals. Rehabilitation is one of the fields benefitting the most from technological development when it comes to activity assessment. A recent review of the past 10-year exercise interventions for people with disabilities reveals that approximately 20% (27/132) of interventions involved technology, ranging from Information and Communication Technologies (ICTs) to devices such as accelerometers and pedometers worn by patients [1]. Study findings demonstrate that technology can be used to conduct tele-exercise sessions with participants in the comfort of their home; deliver Web-based content to a mass audience; measure and monitor activity; and provide fun, interactive and enjoyable forms of movement (i.e., active video games) [1].

Health-related measurement systems are continuously being developed and refined, leading to new opportunities for rehabilitation, such as the possibility to measure three-dimensional movements in various contexts [2]. For instance, an inertial measurement unit (IMU) is a measurement tool combining several triaxial sensors, in most cases an accelerometer, a gyroscope and/or a magnetometer [3, 4]. Other types of sensors can also be added to IMUs to improve or complement the information provided, such as a Global Positioning System (GPS) device [5]. IMUs are small, self-contained and cost-effective devices that may allow acquisition of human movement data (e.g., body posture, upper and lower extremity movements, trunk movements) in an ecological and unconstrained environment [2, 4, 6]. Due to these appealing characteristics, researchers have shown great interest in using these devices as wearable sensors on the human body to analyse multiple daily activities (e.g., walking, running, dressing and eating) [7–10]. Recently, the development of smaller, lower cost and lower power wearable wireless IMUs has increased the possibilities for use in rehabilitation contexts [2, 7]. These technological innovations may allow clinicians to continuously monitor online objective data [11]. Thus, IMUs have the potential to facilitate goal setting and progress monitoring, including effectiveness of the treatment, as well as to support clinical decision-making [2, 11–13]. For instance, objective data collected over a significant period of time can bolster the simple quantitative data collected at limited time-points [2, 11] and information obtained through traditional subjective assessment measures with their inherent limitations related to reliability [2, 13, 14]. Furthermore, as compliance with rehabilitation treatment is often an issue, these objective data can provide patients with possibility to track their own evolution, which may enhance self-motivation and self-efficacy [2, 11].

However, despite the accessibility and potential advantages of IMUs, their uptake and acceptance by health professionals remain a big challenge [15–17]. This is certainly related to the fact that they have mostly been developed considering research and/or engineering requirements without the involvement of clinical end-users [2, 5, 11, 16, 18]. Furthermore, clinical validations of IMU-based systems are rarely conducted [2, 6]. The time required to manage the device and the gathered data is among the main difficulties identified by clinicians when it comes to the use of IMUs [2]. Another major concern is that the data needs to be processed before being clinically usable, which requires a level of technical expertise that clinicians do not necessarily possess [19]. Furthermore, each model of IMU/IMU system has its own specificities, which makes it difficult to use different models of IMUs simultaneously/interchangeably as they may not be compatible. Consequently, it can become cumbersome to combine data from different IMU systems to perform one complete analysis [19]. To facilitate adoption of IMUs by clinicians, collaboration between them and the developers is essential, as perceived ease-of-use and usefulness are key elements to promote successful adoption of such new technologies [2, 20]. Some interdisciplinary teams, such as *Gait Up* [21], *Xsens* [22], *FeetMe* [23] and *Sysnav* [24], have developed systems that are now widely used. However, very few publications describe the use of a user-centered design approach for their process development [25], or this is done only partially or superficially, and very little publications have described rehabilitation professionals’ perceptions and use of such wearable devices [2, 11, 16]. As the uptake of these types of systems depends largely on meeting the needs of end-users, an upstream consultation with clinicians to document their views and insights appears crucial. As with other technological developments in health practices, we first need to understand how end-users (clinicians) feel technology can support their practice. Without such an approach, future adoption and implementation could suffer [26].

It is in this context that our research team is working on the development of IMUs and associated interface for data visualization and analysis specifically designed to be usable and relevant in clinical settings. Given the challenges related to clinical implementation and acceptability of this technology, as well as the potential new opportunities it can provide for clinical practice, it was important for the team to involve clinicians from the get-go, i.e. as early as in the design process [11, 16, 26, 27]. Therefore, the study reported here was first conducted to guide the development of IMUs to ensure their usefulness and use in a clinical physical rehabilitation context, by: 1) exploring clinical needs and potential use of IMUs, and 2) collecting insights on the data analysis and visualization interface designed for the IMUs. To do so, a user-centered design approach was set by encouraging clinicians to highlight potential needs, refinements or issues in implementation, and to express the criteria they would require to maximise implementation and patients’ engagement. In this instance, the clinicians were the end-users the research team wanted to reach.

## Materials and methods

### Study design

We followed a user-centered design method to promote the active participation of key stakeholders, by emphasizing their considerations, needs, concerns and requirements [28]. Such a co-design approach is used to facilitate collaboration between developers and end-users [29]. It also encourages continuous contact with prospective users and uses an iterative process to ensure usability [28].

Focus group methodology was the preferred means of data collection for this study. Focus groups allow participants to share their views among each other on open-ended questions on a topic of common interest [30]. Therefore, focus groups are used to gain information on collective opinions and yield a rich understanding of participants' experiences and beliefs [31]. For the purpose of this study, the research team conducted two series of focus groups: 1) on IMUs and their potential clinical usefulness (objective 1), and 2) on the IMUs data analysis and visualization interface (objective 2).

### Participants

To inform and recruit clinicians, the research team contacted the department heads of three rehabilitation centers in the Montreal area (Canada): 1) *Institut de réadaptation Gingras-Lindsay-de-Montréal* (IRGLM: Institut universitaire sur la réadaptation en déficience physique de Montréal (IURDPM), Centre intégré universitaire de santé et de services sociaux du Centre-Sud-de-l’Île-de-Montréal (CCSMTL)), 2) *Centre de réadaptation Lucie-Bruneau* (CRLB: IURDPM, CCSTML), and 3) *Hôpital juif de réadaptation* (HJR: Centre intégré de santé et de services sociaux de Laval). They were asked to invite their teams to attend a presentation on IMUs. A 30-minute presentation was given by research team members with a healthcare background in the Fall of 2017 to clinicians (n = 24), who were offered to attend either in-person or by video conferencing. The goals of the presentation were to: 1) present the research team and the approach used (user-centered design), 2) familiarize clinicians with IMU-based systems and their components for measuring three-dimensional movements, 3) familiarize clinicians with their potential uses (in the clinic, outdoors and at home), and 4) inform clinicians about the study and of its upcoming two focus groups. Clinicians were then invited to contact the research team if they were interested in taking part in one of the focus groups. Clinicians from a rehabilitation center in Quebec City (Canada), the *Institut de réadaptation en déficience physique de Québec* (IRDPQ: Centre intégré universitaire de santé et de services sociaux de la Capitale-Nationale (CIUSSS-CN)), had gone through a similar process in 2016, as part of a pilot project. Those who had participated in a previous group discussion on IMUs back in 2016 (i.e., the first focus group), which led to the current structured study, were contacted through email to explain the study and the goal of the second series of focus groups, to which they were invited to participate if interested, by contacting the research team back.

In total, clinicians were recruited at four clinical sites in the province of Quebec: IRGLM, CRLB, HJR and IRDPQ. In order to participate in a focus group, clinicians had to: 1) be at least 18 years old, 2) speak French, 3) work with a rehabilitation clientele, and 4) have an interest in using new technologies in their regular clinical practice. Clinicians who were eligible and who had sent an email to express their interest in participating were grouped by site.

### Data collection

All clinicians who took part in the study signed a consent form approved by the local Behavioural Research Ethics Board (CIUSSS-CN #MP-13-2018-453 and RIS_EMP-2017-553). They also had to complete a questionnaire on their occupational profile, which included information on their work experience, clientele they are working with and experience with technologies in their practice, completed by sociodemographic data.

The first series of focus groups was conducted shortly after the general presentations on IMUs at each site. They were facilitated by two research team members who have a healthcare background and who were not directly involved in the technological development of the IMUs. The facilitators started the meetings by making a quick summary of the general presentation on IMUs and asked the participants if they had any questions before proceeding. The focus groups were held in a semi-guided format, following a discussion guide that included potential applications of IMUs to specific clinical problems. A prioritization exercise aimed at identifying the most significant measures for clinical practice was also attempted. S1 Appendix provides the complete discussion guide used for the first series of focus groups. The discussion guide was developed through consultation with the researchers involved in the study to ensure that they would obtain the answers required to guide IMUs’ development. One of the facilitators noted on a board the discussed content and categorized items according to the topics covered, while the other was making sure every question of the guide was covered and that all participants could voice their opinions. Each group discussion lasted about one hour and was audio recorded. It is important to note here that audio recordings from the group discussion conducted in 2016 with clinicians from the IRDPQ were not available. Therefore, no data will be included for this first focus group conducted to this site.

The second series of focus groups was about the interface that could be used for data analysis and visualization. To inform the discussion, a short-narrated video presenting a first version of the software interface was shown to the clinicians at the four sites. The video showed its features and characteristics. After collecting information about the clinicians’ first impressions, the facilitators (the same as for the first series of focus groups) proceeded with the focus group discussion. As for the first series of focus groups, the facilitators followed a discussion guide covering all the topics the developers needed for interface optimization. Clinicians shared their needs and preferences regarding data visualization, analysis, and report format, but also on the overall data presentation (i.e., beyond the interface shown). S2 Appendix provides the complete discussion guide of the second series of focus groups. The discussion guide was also developed through consultation with the researchers involved in the study. Each group discussion lasted about one hour and was audio recorded.

### Data analysis

Descriptive statistics were used to characterize the group of participants (means, standard deviations, response frequencies). Audio recordings from the focus groups were transcribed verbatim in a Word document and analyzed using NVivo qualitative data analysis version 10 software (QSR International Pty Ltd). The study included a total of three verbatim transcripts on IMUs (first series of focus groups) and four on the interface (second series of focus groups), that were coded separately. Two individuals (ÉL, ET and/or ND) performed a thematic analysis [32] by repeatedly reviewing and extracting relevant text from the transcripts and categorizing data into emerging themes and subthemes. More precisely, an early coding template was created to start the data categorization. The coding template was data-driven, which is specific to an inductive approach, and therefore no theoretical framework was used for its design [33]. As the process progressed, the coding guide was adjusted and refined according to the themes and subthemes that were discussed, which is characteristic of a continuous thematic analysis. Any discrepancies were resolved via discussions, and any necessary adjustments were made. For each subtheme that emerged, occurrences, i.e. the number of times the topic was brought up by the participants, were calculated. Percentages, representing the proportion of each subthemes in relation to all the subthemes of a given theme, were also calculated.

## Results

### Participants

Fifteen clinicians took part in the first series of focus groups (clinicians from the IRDPQ site who took part in the 2016 pilot discussion are not taken into account) and 23 in the second one. All participants of the first series took part in the second one. Participants of the first series of focus groups had an average of 16.6±9.6 years of clinical experience. Seven of them (46.7%) had at some point used technological measurement tools in their clinical practice, and only one (6.7%) clearly reported having already used IMUs in the past. Participants of the second series of focus groups had an average of 17.4±9.7 years of clinical experience. Nine of them (39.1%) had at some point used technological measurements tools in their clinical practice, and only one (4.3%) clearly reported having already used IMUs in the past. Table 1 presents demographic and descriptive data in more details.

**Table 1:** Characteristics of the focus groups’ participants.

Characteristics	First series of focus groups (n = 15)		Second series of focus groups (n = 23)	
	n	%	n	%
Rehabilitation center				
CRLB	6	40.0	7	30.4
HJR	5	33.3	6	26.1
IRGLM	4	26.7	5	21.7
IRDPQ	N/A	N/A	5	21.7
Gender				
Woman	13	86.7	20	87.0
Man	2	13.3	3	13.0
Age (years)				
20–29	3	20.0	4	17.4
30–39	3	20.0	5	21.7
40–49	6	40.0	9	39.1
50–59	3	20.0	5	21.7
Clinical practice area				
Physical therapist	10	66.7	15	65.2
Occupational therapist	4	26.7	7	30.4
Physical rehabilitation technician	1	6.7	1	4.3
Main clientele^a^				
Stroke	4	17.4	8	19.5
Brain injury	2	8.7	4	9.8
Multiple sclerosis	2	8.7	3	7.3
Spinal cord injury	6	26.1	10	24.4
Amputation	2	8.7	5	12.2
Others	7	30.4	11	26.8

^a^ Possibility of more than one answer per participant.

### Categories of variables to measure and characteristics of IMUs

Relevant text from the verbatim transcripts of the first series of focus groups was categorized into two main themes: 1) categories of variables that would be useful to measure with IMUs in clinical practice, and 2) desired characteristics of the IMUs.

#### Categories of variables to measure with IMUs

Clinical needs that could be addressed with IMUs were varied and site dependent. Clinicians identified ten main categories of variables that would be useful to measure with IMUs in clinical practice: 1) gait (n = 24 occurrences; 24.0%), 2) posture (n = 18; 18.0%), 3) activities (n = 16; 16.0%), 4) falls and losses of balance (n = 8; 8.0%), 5) muscular activity (n = 7; 7.0%), 6) limbs orientation (n = 6; 6.0%), 7) physiological data (n = 6; 6.0%), 8) compensations (n = 5; 5.0%), 9) wheelchair use (n = 5; 5.0%), and 10) movement quality (n = 5; 5.0%). Table 2 presents responses of clinicians from each of the three sites, with some examples of items discussed for each identified variable. These examples are mainly summarized quotations or ideas, expressed by one or many participants, that illustrated each subtheme.

**Table 2:** Categories of variables that would be useful to measure with IMUs in clinical practice.

Categories of variables	Examples of items discussed	Occurrences			TOTAL (n = 100)	
		IRGLM	CRLB	HJR	n	%
Gait	Average speed and variations in speed, distance covered, number of steps, walking pattern, step length, lower limb movement symmetry, range of motion	7	10	7	24	24.0
Posture	Space occupied, micro-movements, dynamic vs static postures, upper body alignment, dynamic weight-bearing, dysmetria	7	8	3	18	18.0
Activities	% or duration of activity (activity level), night activity (sleepwalking), use of a limb (hemiplegia, symmetry), lying down/standing (number of times, complete or incomplete), activity bouts	5	2	9	16	16.0
Falls and losses of balance	Number of falls and losses of balance, pre-fall postural reactions	0	1	7	8	8.0
Muscular activity	Electromyogram, eccentric vs concentric, muscle balance, muscle fatigue, muscle contraction intensity	2	5	0	7	7.0
Limbs orientation	Angles, range of motion, movement in space, time spent above a certain elevation angle, body orientation	1	3	2	6	6.0
Physiological data	Saturation (O_2_), blood pressure, heart rate, respiration	3	0	3	6	6.0
Compensations	Types of compensations (top of the body turns too much on one side, decreased arm swing on one side, foot inwards), degree of compensation	0	5	0	5	5.0
Wheelchair use	Number of propulsions, changes in buttock pressure, distance covered, number of falls	3	1	1	5	5.0
Movements quality	Tremors, spasms, oscillations, fluidity	2	2	1	5	5.0

#### Desired characteristics of the IMUs

According to participants, a clinically usable IMU system needs to: 1) be easy to install/uninstall (n = 12 occurrences; 25.0%), 2) display some parameters/feedback when in operation (n = 10; 20.8%), 3) be easy to wear for the patient (n = 10; 20.8%), 4) be easy to use (n = 8; 16.7%), 5) be sturdy (n = 5; 10.4%), and 6) be affordable (n = 3; 6.3%). Table 3 presents clinicians’ responses from each of the three sites, with some examples of items discussed for each desired characteristic.

**Table 3:** Desired characteristics of the IMUs.

Desired characteristics	Examples of items discussed	Occurrences			TOTAL (n = 48)	
		IRGLM	CRLB	HJR	n	%
Easy to install/ uninstall	Simple and quick installation/ uninstallation	2	8	2	12	25.0
Displayed parameters	Biofeedback, battery life, data collection duration, data recording, light indicating that the device is working	2	6	2	10	20.8
Easy to wear	Discreet, lightweight, comfortable, stable, not cumbersome, difficult to remove so as not to lose it	2	3	5	10	20.8
Easy to use	Ease of generating results, fast to use, practical, provides concrete information, few sensors, not too many control buttons	6	1	1	8	16.7
Sturdy	Waterproof, cold resistant, washable	2	3	0	5	10.4
Affordable	Cost-effective, affordable	1	0	2	3	6.3

### IMUs data analysis and visualization interface

Relevant text from the verbatim transcripts of the second series of focus groups was categorized into fours main themes: 1) functionalities, 2) display options, 3) clinical data and associated information, and 4) data collection duration.

#### Functionalities

Clinicians expressed their thought regarding the functionalities that the interface must present to meet their needs. Those most frequently mentioned were: 1) speed of results generation (n = 24 occurrences; 28.9%), and 2) flexibility of raw data transformation (n = 13; 15.7%). Table 4 presents clinicians responses from each of the four sites, with some examples of items discussed for each functionality.

**Table 4:** Functionalities.

Functionalities	Examples of items discussed	Occurrences				TOTAL (n = 83)	
		IRGLM	CRLB	HJR	IRDPQ	n	%
Speed of results generation	Very few "clicks"/ minutes between loading the data and obtaining the report	6	4	5	9	24	28.9
Flexibility of raw data transformation	Possibility to select the desired raw data and to apply filters (checkboxes with options of filters)	2	5	1	5	13	15.7
Software compatibility with various devices	Desktop, laptop, tablet, accessible/ installed on several devices/workstations	1	4	4	0	9	10.8
Printed or PDF report	To easily attach the report to the patient’s file	3	2	2	1	8	9.6
Joint angles measurements a posteriori	Recording and calculation of joint angles from data selected a posteriori	0	5	0	3	8	9.6
File formats and size	Files/reports must be light due to issues with keeping/sharing large files	1	0	4	1	6	7.2
Visualization of several measurement times on the screen	Progress monitoring, follow-up, pre-post, date selection	0	3	0	2	5	6.0
Interface customizability	Favorite list, user profile	2	1	2	0	5	6.0
Gait sequence analysis	Possibility to sequence the walking pattern	0	2	0	2	4	4.8
Visualization of data in video format	Possibility to view data in video format directly within the interface	0	1	0	0	1	1.2

#### Display options

Several display options related to data visualization were discussed by clinicians, including: 1) simultaneous visualization of several measurement times on the screen (n = 9 occurrences; 16.4%), 2) presentation of comparisons (n = 9; 16.4%), and 3) processed data presentation (n = 9; 16.4%). Table 5 presents responses of clinicians from each of the four participating rehabilitation centers, with some examples of items discussed for each display options.

**Table 5:** Display options.

Display options	Examples of items discussed	Occurrences				TOTAL (n = 55)	
		IRGLM	CRLB	HJR	IRDPQ	n	%
Simultaneous visualization of several measurement times on the screen	Possibility to display different measurement times for a same variable (progress monitoring, when there is no normative data)	1	4	3	1	9	16.4
Presentation of comparisons	Visually highlighting comparisons using different colors, superposition, side by side positioning	1	7	0	1	9	16.4
Processed data presentation	Presentation of scores (rather than the raw data)	4	3	2	0	9	16.4
Visualization of the difference between results and normative data	Visually highlighting the difference between the results and the normative data	2	2	0	3	7	12.7
Results display options	Choices of filters (checkboxes), predefined data list, possibility to choose the information/results that appears or not	1	2	1	2	6	10.9
Simplified data presentation	To facilitate patients’ understanding, to facilitate interpretation of results	2	0	2	2	6	10.9
Visualization of normative data	Normative data clearly visible/ highlighted, options on how the normative data is shown (a line, a different color, an avatar, etc.)	2	2	0	1	5	9.1
Types of graphs	Visually simple graphs, e.g., bar graphs, circular-arc graphs	0	2	2	0	4	7.3

#### Clinical data reported and associated information

Participants identified several clinical data and some associated information they would like to visualize in the report generated using the interface. Many of them mentioned pre-post results (n = 19 occurrences; 15.3%), gait/walking pattern (n = 16; 12.9%), filtered data (n = 13; 10.5%), normative data (n = 3; 10.5%), and joint angle measurements (n = 13; 10.5%). Table 6 presents responses of clinicians from each of the four sites.

**Table 6:** Clinical data and associated information.

Clinical data and associated information	Occurrences				TOTAL(n = 124)	
	IRGLM	CRLB	HJR	IRDPQ	n	%
Pre-post results and comparative curves	2	7	3	7	19	15.3
Gait/walking pattern	4	4	0	8	16	12.9
Filtered data	4	4	3	2	13	10.5
Normative data	2	5	0	6	13	10.5
Joint angles measurement	0	5	0	8	13	10.5
Speed	0	2	2	4	8	6.5
Weight-bearing or buttock pressure	0	4	2	1	7	5.6
Real-time visualization/immediate results presentation	0	2	0	3	5	4.0
Activity time	3	1	1	0	5	4.0
Biofeedback to the patients (in real time)	0	0	3	2	5	4.0
Multi-segment coordination	1	1	0	3	5	4.0
Specific periods of time/sequences	1	1	2	1	5	4.0
Deviation from the clinical goal	1	0	0	2	3	2.4
Mean or median	0	2	0	1	3	2.4
Raw data	0	0	0	3	3	2.4
Muscular strength	0	1	0	0	1	0.8

#### Data collection duration

Respones related to data collection duration wanted with IMUs ranged from a few minutes to one week. Table 7 presents responses of clinicians from each of the four sites.

**Table 7:** Data collection duration.

Data collection duration	Occurrences				TOTAL (n = 12)	
	IRGLM	CRLB	HJR	IRDPQ	n	%
One day	2	2	2	0	6	50.0
One week	0	2	1	0	3	25.0
More than one day, but less than one week	0	0	2	0	2	16.7
A few minutes (during a session)	0	0	1	0	1	8.3

### Influence of IMUs on clinical practice and added value

The potential usefulness and influence of IMUs on clinical practice was addressed by clinicians in both series of focus groups. They discussed two main aspects. First, this technology could allow them to establish a quantitative profile of activities performed outside of the care/rehabilitation sessions (e.g., use of limb(s) on the affected side by hemiplegic patients, community/home walking pattern, falls or loss of balance, number of occurrences of a non-recommended movement, return to work following an injury). Second, such systems could allow them to gather quantitative data related to variables that are normally difficult to assess due to their complexity (e.g., gait). According to the participants, this would be useful at follow-up, to discuss progress and compliance with patients. It could also provide patients with motivation and assist self-management.

Clinicians also mentioned several criteria that would be added values to the system and its interface so that they would be more likely to use it. Among these, they mentioned ease of use (n = 7 occurrences; 21.2%), short analysis time (n = 4; 12.1%) and system validation (n = 3; 9.1%). Table 8 presents responses of clinicians from each of the four sites.

**Table 8:** Added value.

Added value	Occurrences				TOTAL (n = 33)	
	IRGLM	CRLB	HJR	IRDPQ	n	%
Ease of use	2	2	1	2	7	21.2
Short analysis time	1	1	1	1	4	12.1
System validation	0	2	0	1	3	9.1
Training course	0	1	0	1	2	6.1
User guide (video/2-page)	0	2	0	0	2	6.1
Human assistance	0	1	1	0	2	6.1
System reliability	0	2	0	0	2	6.1
More efficient than commercial software	0	0	2	0	2	6.1
Cost	0	0	1	1	2	6.1
Accuracy of data/results	1	0	0	1	2	6.1
Complement to the standard evaluation	2	0	0	0	2	6.1
System flexibility (measures and results)	0	0	1	0	1	3.0
Easily readable writing (large characters)	0	0	1	0	1	3.0
Computers accessibility	0	0	0	1	1	3.0

## Discussion

This study explored clinical needs and potential use of IMU-based systems in clinical practice, and collected feedback on the data analysis and visualization interface designed by our team for the IMUs. Regarding the use of IMUs, clinicians expressed an interest, had a good understanding of the broad spectrum of possible uses, and articulated needs that were diverse and based on specific measures. However, their expectations in terms of characteristics of IMUs, data processing and data visualization were very high before considering using IMUs in their clinical practice. Our results showed that they wanted a reliable and valid system, in which they could have confidence, as also suggested by Schall et al. [34]. This finding is perfectly legitimate. The system must bring something more than the tools or systems they already use, while still being easy to use. It must also generate results and reports that are visually easy to interpret. In both series of focus groups, clinicians mentioned many aspects related to categories of variables to measure with IMUs, characteristics of IMUs, data analysis and visualization interface to ensure clinical usefulness of IMUs and their added value. This is in accordance with the Technology Acceptance Model, which states that the level of acceptability of technologies depends mainly on the users’ perceived ease of use and perceived usefulness [20].

Despite a wide variety of needs expressed, measure and analysis of gait were largely discussed by many of the clinicians who took part in this study. They argued that this is a very complex variable that involves a multitude of related sub-variables (e.g., weight-bearing, multi-segment coordination, joint angles excursion, symmetry). As they normally based their assessment on simple quantitative data (e.g., walking distance, walking speed, static muscular strength) and on qualitative/subjective observations (e.g., movement quality), they clearly stated that IMUs could be useful to enrich this variable. Interestingly, a recent study conducted by Bernhard et al. [35] showed the feasibility of assessing gait and balance features with IMUs in clinical settings for neurological patients. If properly implemented, clinicians from the current study saw also the IMUs’ potential to monitor objectively activities performed outside of the care/rehabilitation settings. Indeed, current clinical practice offers few opportunities in this regard [11]. Similarly, they discussed their potential to document remotely objective outcome measures and to promote compliance to treatment and self-motivation, which have been shown to be possible with IMUs [36, 37]. This is in accordance with the literature that documented perspectives of health professionals on wearable technologies and their added value for assessment and intervention [2, 11, 38].

We believe that the development of IMUs that meet needs clearly expressed by clinicians will provide them with new opportunities in their clinical practice. Like several other studies, our work highlights the importance of a user-centered design approach and of a close collaboration between engineers, researchers and end-users to promote IMUs acceptance [11, 16, 26, 27, 39, 40]. Developers cannot assume what the clinicians’ needs and preferences are [28, 29]. Furthermore, considering and addressing barriers, facilitators and concerns of clinicians and of clinical settings (e.g., time, financial and material resources) is warranted [34]. This study identified several important aspects and features to promote the appropriation of IMUs by clinicians, which may inform the development of future IMU-based technologies for rehabilitation. As the actual end-users of such systems were consulted in an upstream process, this study is an added value to the existing literature. Indeed, studies exploring clinicians’ perspectives on IMUs and their visualization interface prior to their development are missing.

As part of the first series of focus groups, a prioritization exercise of the variables that would be most useful to measure with IMUs in clinical practice was attempted. Indeed, responses were very different depending on the rehabilitation center, the clinical program and the participants’ experience and expertise. As relevant technologies continue to improve, and considering the increased possibilities for use in rehabilitation contexts, it is important to deepen the discussion over variables that are most significant for clinical practice, including a prioritization exercise. In the near future, an online survey will be developed on the basis of the results of this study to obtain the opinion of a larger number of clinicians and clinical settings. Such an identification and prioritization process will provide insights to inform IMU systems design that will correspond to health professionals’ expectations and needs, and could therefore contribute to promote better uptake and acceptance. This will be the subject of a separate paper. A mixed methods approach is recommended when conducting a study that focuses on a multifaceted or complex phenomenon, as in the case of a user-centered design [41]. Meaningful integration of qualitative and quantitative techniques when performing user-centred design allows to collect rich and comprehensive data, and to reflect more accurately end-users’ perspectives [42]. The data can be compared and combined, thus providing a more complete overview and understanding of their needs and priorities [42, 43]. The survey results will therefore complement the data collected through the focus groups. It will also be an opportunity for clinicians, researchers and developers to interact with each other, and to conduct a participatory research project [44]. Assessing IMUs’ usability with clinicians and rehabilitation patients, two end-user groups with different needs, with a prototype that meets most of their needs and includes a basic interface is a logical next step to explore key aspects of technology uptake such as efficiency, effectiveness and satisfaction [45]. Real-world validation, which is often missing, needs also to be performed [4].

This study is not without some limitations. Time constraints for focus groups (i.e., maximum 60 minutes per focus group) made it difficult to deepen the discussion regarding implementation and confidence in use of the technology as well as the rationale behind the participants’ answers, and to prioritize the characteristics that were brought up. Although it was decided not to present an IMU system prototype to clinicians prior to the focus groups in order not to bias their thinking, this choice made it harder for them to visualize the use of the developed technology (i.e., how and in which context). In contrast, showing them a short-narrated video presenting a first version of an interface may have biased or influenced their thoughts and comments. Presentations before both rounds of focus groups may have limited participants’ perspectives, but were necessary for those who were unfamiliar with IMUs and for all to have the same understanding. Because of the inclusion criteria, the rehabilitation professionals who took part in the study had an interest in using new technologies in their regular clinical practice. This might explain why we did not get any response that IMUs were perceived as useless. Due to specificities of clinicians involved in this study (e.g., influence of the rehabilitation center, clinical program and background), we obtained individual perceptions: a very limited number of participants were sharing the same vision. Part of this can be attributed to the small sample size. Therefore, our results cannot be directly generalized to other practice settings. In addition, some health professionals were not represented, such as physiatrists, while some health professionals were over-represented, such as physical therapists. However, in Canada, clinicians who perform assessments related to movement analysis, including gait analysis, are mostly physical therapists. Also, the clinicians all came from institutions with similar practices and cultures, thus supporting the need for a large survey designed for a greater number of health professionals working in different contexts. Finally, rehabilitation patients were not included in this study. We consider that they could offer a unique perspective that could be the subject of a separate study.

## Conclusions

Despite the accessibility and potential advantages of IMUs, their uptake and acceptance by clinicians remain a big challenge. Thus, through a user-centered design approach and a focus group methodology, we conducted a study to help guide the development process of IMUs to ensure their usefulness and use in the clinical context. We gathered clinicians’ views on categories of variables that would be useful to measure with IMUs in clinical practice, characteristics they should present, and their potential influence on clinical practice. We also collected information on data analysis and visualization interface needs (i.e., functionalities, display options, clinical data reported, data collection duration). Clinicians expressed positive opinions regarding the clinical use of IMUs, but their expectations were high before considering using IMUs in their practice. The lack of consensus around prioritization of development indicates that more interdisciplinary concertations should be conducted. Our hope is that the results from this study will improve IMU systems development and increase the functionality and applicability of IMUs in clinical contexts, as these systems have the potential to facilitate goal setting and progress monitoring, to support clinicians’ decision-making and to provide patients with the motivation to achieve clinical goals.

## Supporting information

S1 Appendix Discussion guide of the first series of focus groups. (DOCX)

S2 Appendix Discussion guide of the second series of focus groups. (DOCX)

## References

[1] Lai B, Young H-J, Bickel CS, Motl RW, Rimmer JH. Current trends in exercise intervention research, technology, and behavioral change strategies for people with disabilities: A scoping review. Am J Phys Med Rehabil. 2017; 96(10): 748–761. 10.1097/PHM.0000000000000743 28398967

[2] Papi E, Murtagh GM, McGregor AH. Wearable technologies in osteoarthritis: A qualitative study of clinicians' preferences. BMJ Open. 2016; 6(1): e009544 10.1136/bmjopen-2015-009544 26810998 PMC4735169

[3] Madgwick SO, Harrison AJ, Vaidyanathan R. Estimation of IMU and MARG orientation using a gradient descent algorithm. IEEE Int Conf Rehabil Robot. 2011; 2011: 5975346 10.1109/ICORR.2011.5975346 22275550

[4] O'Reilly M, Caulfield B, Ward T, Johnston W, Doherty C. Wearable inertial sensor systems for lower limb exercise detection and evaluation: A systematic review. Sports Med. 2018; 48(5): 1221–1246. 10.1007/s40279-018-0878-4 29476427

[5] Chen L, Hu H. IMU/GPS based pedestrian localization. In: Proc 4th Computer Science & Electronic Engineering Conf; 2012 Sept 12–13; Colchester, UK. p. 23–28.

[6] Wang Q, Markopoulos P, Yu B, Chen W, Timmermans A. Interactive wearable systems for upper body rehabilitation: A systematic review. J Neuroeng Rehabil. 2017; 14(1): 20 10.1186/s12984-017-0229-y 28284228 PMC5346195

[7] Valero E, Sivanathan A, Bosché F, Abdel-Wahab M. Musculoskeletal disorders in construction: A review and a novel system for activity tracking with body area network. Appl Ergon. 2016; 54: 120–130. 10.1016/j.apergo.2015.11.020 26851471

[8] Novak D, Goršič M, Podobnik J, Munih M. Toward real-time automated detection of turns during gait using wearable inertial measurement units. Sensors. 2014; 14(10): 18800–18822. 10.3390/s141018800 25310470 PMC4239865

[9] Luinge HJ, Veltink PH, Baten CTM. Ambulatory measurement of arm orientation. J Biomech. 2007; 40: 78–85. 10.1016/j.jbiomech.2005.11.011 16455089

[10] Vanveerdeghem P, Van Torre P, Stevens C, Knockaert J, Rogier H. Synchronous wearable wireless body sensor network composed of autonomous textile nodes. Sensors. 2014; 14(10): 18583–18610. 10.3390/s141018583 25302808 PMC4239931

[11] Argent R, Slevin P, Bevilacqua A, Neligan M, Daly A, Caulfield B. Clinician perceptions of a prototype wearable exercise biofeedback system for orthopaedic rehabilitation: A qualitative exploration. BMJ Open. 2018; 8(1): e026326 10.1136/bmjopen-2018-026326 30366919 PMC6224760

[12] Caulfield BM, Donnelly SC. What is connected health and why will it change your practice? QJM. 2013; 106(8): 703–707. 10.1093/qjmed/hct114 23676416

[13] Carnevale A, Longo UG, Schena E, Massaroni C, Presti DL, Berton A, et al Wearable systems for shoulder kinematics assessment: a systematic review. BMC Musculoskelet Disord. 2019; 20(546). 10.1186/s12891-019-2930-4 PMC6858749 31731893

[14] Bassett SF. The assessment of patient adherence to physiotherapy rehabilitation. New Zeal J Physiother. 2003; 31(2): 60–66.

[15] Shull PB, Jirattigalachote W, Hunt MA, Cutkosky MR, Delp SL. Quantified self and human movement: A review on the clinical impact of wearable sensing and feedback for gait analysis and intervention. Gait Posture. 2014; 40(1): 11–19. 10.1016/j.gaitpost.2014.03.189 24768525

[16] Bergmann JHM, McGregor AH. Body-worn sensor design: what do patients and clinicians want? Ann Biomed Eng. 2011; 39(9): 2299–2312. 21674260 10.1007/s10439-011-0339-9

[17] Appelboom G, Camacho E, Abraham ME, Bruce SS, Dumont EL, Zacharia BE, et al Smart wearable body sensors for patient self-assessment and monitoring. ArchPublic Health. 2014; 72(1): 28. 10.1186/2049-3258-72-28 PMC4166023 25232478

[18] Blumenthal J, Wilkinson A, Chignell M. Physiotherapists’ and physiotherapy students’ perspectives on the use of mobile or wearable technology in their practice. Physiother Can. 2018; 70(3): 251–261. 10.3138/ptc.2016-100.e 30275650 PMC6158559

[19] Espay AJ, Bonato P, Nahab FB, Maetzler W, Dean JM, Klucken J, et al Technology in Parkinson's disease: Challenges and opportunities. Mov Disord. 2016; 31(9): 1272–1282. 10.1002/mds.26642 27125836 PMC5014594

[20] Davis FD. Perceived usefulness, perceived ease of use, and user acceptance of information technology. Mis Quart. 1989; 13(3): 319–340.

[21] Gait Up [Internet]. Gait Up; c2013-2020 [cited 2020 June 23]. Available from: https://gaitup.com/.

[22] Xsens [Internet]. Xsens; [cited 2020 June 23]. Available from: https://www.xsens.com/.

[23] FeetMe [Internet]. FeetMe; c2020 [cited 2020 June 23]. Available from: https://feetme.fr/en.

[24] Sysnav [Internet]. Sysnav; c2020 [cited 2020 June 23]. Available from: https://www.sysnav.fr/?lang = en.

[25] Klaassen B, van Beijnum B-J, Weusthof M, Hofs D, van Meulen F, Drooget E, et al A Full Body Sensing System for Monitoring Stroke Patients in a Home Environment In: Plantier G, Schultz T, Fred A, Gamboa H, editors. Biomedical Engineering Systems and Technologies. BIOSTEC 2014. Communications in Computer and Information Science. Cham: Springer; 2015; 511.

[26] Shah SG, Robinson I. Benefits of and barriers to involving users in medical device technology development and evaluation. Int J Technol Assess Health Care. 2007; 23(1): 131–137. 10.1017/S0266462307051677 17234027

[27] Michie S, Yardley L, West R, Patrick K, Greaves F. Developing and evaluating digital interventions to promote behavior change in health and health care: Recommendations resulting from an international workshop. J Med Internet Res. 2017; 19(6): e232 10.2196/jmir.7126 28663162 PMC5509948

[28] Baek E-O, Cagiltay K, Boling E, Frick T. User-centered design and development In: Spector JM, Merrill MD, van Merriënboer J, Driscoll MP, editors. Handbook of research on educational communications and technology, 3rd ed Abingdon-on-Thames, UK: Routledge; 2008 p. 660–668.

[29] Sanders E B-N, Westerlund B. Experiencing, exploring and experimenting in and with co-design spaces. In Proc Nordic Design Research Conf; 2011 May 29–31; Helsinki, Finland.

[30] Kitzinger J. Qualitative research. Introducing focus groups. BMJ. 1995; 311: 299–302. 10.1136/bmj.311.7000.299 7633241 PMC2550365

[31] Morgan DL. The Focus Group Guidebook. Thousand Oaks, CA: Sage Publications; 1998.

[32] Guest G, MacQueen KM, Namey EE. Applied Thematic Analysis. Thousand Oaks, CA: Sage Publications; 2012.

[33] Nowell LS, Norris JM, White DE, Moules NJ. Thematic analysis: Striving to meet the trustworthiness criteria. Int J Qual Methods. 2017; 16(1): 1–13.

[34] Schall MC Jr, Sesek RF, Cavuoto LA. Barriers to the adoption of wearable sensors in the workplace: A survey of occupational safety and health professionals. Hum Factors. 2018; 60(3): 351–362. 10.1177/0018720817753907 29320232 PMC9307130

[35] Bernhard FP, Sartor J, Bettecken K, Hobert MA, Arnold C, Weber YG, et al Wearables for gait and balance assessment in the neurological ward—study design and first results of a prospective cross-sectional feasibility study with 384 inpatients. BMC Neurol. 2018; 18(1): 114 10.1186/s12883-018-1111-7 30115021 PMC6094895

[36] Argent R, Daly A, Caulfield B. Patient involvement with home-based exercise programs: Can connected health interventions influence adherence? JMIR Mhealth Uhealth. 2018; 6(3): e47 10.2196/mhealth.8518 29496655 PMC5856927

[37] Cooper G, Sheret I, McMillan L, Siliverdis K, Sha N, Hodgins D, et al Inertial sensor-based knee flexion/extension angle estimation. J Biomech. 2009; 42(16): 2678–2685. 10.1016/j.jbiomech.2009.08.004 19782986

[38] Sprint G, Cook DJ, Weeks DL. Designing Wearable Sensor-Based Analytics for Quantitative Mobility Assessment. IEEE Int Conf Smart Comput SMARTCOMP. 2016.

[39] Baig MM, GholamHosseini H, Moqeem AA, Mirza F, Lindén M. A systematic review of wearable patient monitoring systems—Current challenges and opportunities for clinical adoption. J Med Syst. 2017; 41(7): 115 10.1007/s10916-017-0760-1 28631139

[40] Klaassen B, van Beijnum BF, Held JP, Reenalda J, van Meulen FB, Veltink PH, et al Usability evaluations of a wearable inertial sensing system and quality of movement metrics for stroke survivors by care professionals. Front Bioeng Biotechnol. 2017; 5: 20 10.3389/fbioe.2017.00020 28421180 PMC5377936

[41] Alwashmi MF, Hawboldt J, Davis E, Fetters MD. The iterative convergent design for mobile health usability testing: Mixed methods approach. JMIR Mhealth Uhealth. 2019; 7(4): e11656 10.2196/11656 31025951 PMC6658163

[42] Wisdom J, Creswell JW. Mixed Methods: Integrating quantitative and qualitative data collection and analysis while studying patient-centered medical home models. Rockville, MD: Agency for Healthcare Research and Quality; 2013.

[43] Fetters MD, Curry LA, Creswell JW. Achieving integration in mixed methods designs–Principles and practices. Health Serv Res. 2013; 48(6 Pt 2): 2134–2156. 10.1111/1475-6773.12117 24279835 PMC4097839

[44] Cargo M, Mercer SL. The value and challenges of participatory research: Strengthening its practice. Annu Rev Public Health. 2008; 29, 325–350. 10.1146/annurev.publhealth.29.091307.083824 18173388

[45] International Organisation for Standardisation. ISO 9241–11 Ergonomics of Human-System Interaction-Part 11: Usability: Definitions and Concepts. Geneva, Switzerland: International Organisation for Standardisation; 2018.

