# Supplementary material for: Clinicians’ perspectives on inertial measurement units in clinical practice
Source: PLoS One. 2020 Nov 13;15(11):e0241922. doi: 10.1371/journal.pone.0241922 (PMC7665628; doi:10.1371/journal.pone.0241922)
Supplement: S1 Appendix — (DOCX) [file pone.0241922.s001.docx]

S1 Appendix. Discussion guide of the first series of focus groups.

1. **General introductory discussion**

*Question:*

- 1. Please introduce yourself, share about your clinical practice (the clients you see in your practice) and describe your role.

1. **Brief reminder of the presentation on motion measurement and inertial measurement units (including examples of use)**
2. **Discussion about potential application of the technology to specific clinical problems**

*Questions:*

- 1. What are your first impressions regarding this technology? (Clarification questions can be answered by the team member presenting the technology)
  2. What should have/contain an inertial measurement unit to meet your needs?
  3. What should have/contain an inertial measurement unit to be usable in a clinical context?
  4. What data would you need to measure and analyze:
- *In clinic?*
- *In training session?*
- *At home?*
- *In the community*?

(The facilitator will note all answers on a board. Responses will be grouped if appropriate. The facilitator will give post-it notes to participants.)

- 1. Reflecting on all ideas that have been discussed, please place a post-it note next to the two clinical measures that you find most relevant for your practice.

1. **Discussion to determine which elements of the practice are influenced by technology**

*Question:*

- 1. What element of your practice, related to motion measurement, would you change that would have a significant impact on the service provided or the access to care?

1. **Discussion to determine what should be included in a clinically usable and useful analysis report**

*Question:*

- 1. What do you want to see in an analysis report?
