# Supplementary material for: Clinicians’ perspectives on inertial measurement units in clinical practice
Source: PLoS One. 2020 Nov 13;15(11):e0241922. doi: 10.1371/journal.pone.0241922 (PMC7665628; doi:10.1371/journal.pone.0241922)
Supplement: S2 Appendix — (DOCX) [file pone.0241922.s002.docx]

**S2 Appendix. Discussion guide of the second series of focus groups.**

1. **Introduction**

*Questions:*

- 1. Have any of you ever used, or are you currently using, inertial measurement units and their analysis software? If yes:
     1. Which sensor(s) do you use/have you used?
     2. Which features/functions of the software currently in use are most useful?
     3. Which features/functions of the software currently in use do not meet your needs?
     4. Do you know any algorithms/reports that you would like to see implemented?

1. **Presentation of the interface at the current stage of its development and discussion on the potential use of the technology**

*Questions:*

- 1. What are your first impressions regarding the presented interface? Are there any elements of the interface that you would change?
  2. What should have/contain the interface to meet your needs?
  3. What should have/contain the interface or the report to be usable in a clinical context?
  4. What clinical data would you like to visualize in the report generated using the interface?
     1. Data/results in the form of number(s) vs signals?
     2. Functional data vs participation data?
     3. For which body members would you like to visualize the clinical data? Single segment or multi-segment?
     4. Collection time (one hour, one day, one week)?
     5. Time to consult the results/data obtained?
  5. How many "clicks" would you be ready to make between loading of your data and obtaining the report?
  6. How would you use the data in your clinical practice?
     1. Which platform(s) would you use for visualization of data/analysis/reports (PC, tablet, cell phone)? (For you, with patients, for files?)
        1. What measures should be inferred from the data?
  7. What would make you trust the system in front of you?
     1. What is the main factor that would determine the choice of one analysis software over another (costs, ease of use, reliability, quality of reports, types of analyses)?
